# Mining of long non-coding RNAs with target genes in response to rust based on full-length transcriptome in Kentucky bluegrass

**DOI:** 10.3389/fpls.2023.1158035

**Published:** 2023-05-09

**Authors:** Xueying Zhao, Xiaoyang Sun, Yang Chen, Hanfu Wu, Yujiao Liu, Yiwei Jiang, Fuchun Xie, Yajun Chen

**Affiliations:** ^1^ College of Horticulture, Northeast Agricultural University, Harbin, China; ^2^ College of Animal Science and Technology, Northeast Agricultural University, Harbin, China; ^3^ College of Life Science, Agriculture and Forestry, Qiqihar University, Qiqihar, China; ^4^ Department of Agronomy, Purdue University, West Lafayette, IN, United States

**Keywords:** full-length transcriptome, lncRNAs, *Poa pratensis*, rust, resistance mechanism

## Abstract

Kentucky bluegrass (*Poa pratensis* L.) is an eminent turfgrass species with a complex genome, but it is sensitive to rust (*Puccinia striiformis*). The molecular mechanisms of Kentucky bluegrass in response to rust still remain unclear. This study aimed to elucidate differentially expressed lncRNAs (DELs) and genes (DEGs) for rust resistance based on the full-length transcriptome. First, we used single-molecule real-time sequencing technology to generate the full-length transcriptome of Kentucky bluegrass. A total of 33,541 unigenes with an average read length of 2,233 bp were obtained, which contained 220 lncRNAs and 1,604 transcription factors. Then, the comparative transcriptome between the mock-inoculated leaves and rust-infected leaves was analyzed using the full-length transcriptome as a reference genome. A total of 105 DELs were identified in response to rust infection. A total of 15,711 DEGs were detected (8,278 upregulated genes, 7,433 downregulated genes) and were enriched in plant hormone signal transduction and plant–pathogen interaction pathways. Additionally, through co-location and expression analysis, it was found that lncRNA56517, lncRNA53468, and lncRNA40596 were highly expressed in infected plants and upregulated the expression of target genes *AUX/IAA*, *RPM1*, and *RPS2*, respectively; meanwhile, lncRNA25980 decreased the expression level of target gene *EIN3* after infection. The results suggest that these DEGs and DELs are important candidates for potentially breeding the rust-resistant Kentucky bluegrass.

## 1 Introduction

Kentucky bluegrass (*Poa pratensis* L.) is one of the most dominant cool-season grass species (Saud et al., 2017). However, rust [*Puccinia striiformis*] infection has become increasingly prevalent in certain cultivars of this species (Beirn et al., 2011). *P. striiformis* is an obligate biotrophic fungi that grows and reproduces only on living host plant tissues (Lu et al., 2016). Rust symptoms on Kentucky bluegrass typically appear as yellow- or orange-colored foliage (Beirn et al., 2015). The infected plants die out completely, owing to the decrease of the photosynthetic rate and the increase of dark respiration (Beirn et al., 2011). In zoysiagrass (*Zoysia japonica*), the rust pathogen was identified as *Puccinia zoysiae*, and the molecular mechanisms related to rust resistance were explored using transcriptome sequencing (Zhang et al., 2022). However, knowledge on the resistance mechanisms of Kentucky bluegrass against rust currently remains limited.

At the molecular level, plants gradually evolve a variety of elaborate and effective defense strategies to resist pathogen invasion (Jones and Takemoto, 2004; Nejat et al., 2017; Dong et al., 2020). The plant immune system is broadly divided into effector-triggered immunity (ETI) and pathogen-associated molecular pattern-triggered immunity (PTI) (Jones and Dangl, 2006). In PTI, the biological functions of CDPKs are often closely related to various hormone signaling pathways, including ethylene, auxin, salicylic acid (SA), and jasmonic acid (JA) (Ludwig et al., 2005; Coca and San Segundo, 2010). In grapevine (*Vitis vinifera* L.), overexpression of two *VpCDPK* genes enhanced powdery mildew (PM) resistance *via* positively regulating ethylene and SA signaling (Hu et al., 2021a). In ETI, resistance (*R*) genes are the most effective weapons against invasion *via* recognizing the corresponding pathogen effectors (Nimchuk et al., 2003; Zhao et al., 2022). Some evidence has shown that *R* genes increase rust resistance in grain crop species (Wang et al., 2020; Chen et al., 2022; Dinh et al., 2022). For example, *RppK* gene and its cognate gene *AvrRppK* gene significantly enhanced resistance against *Puccinia polysora* in maize (*Zea mays* L.) (Chen et al., 2022). *TaRPM1* gene positively regulated the resistance to *Puccinia striiformis* f. sp. *tritici* (*Pst*) through the SA signaling pathway in wheat (*Triticum aestivum* L.) (Wang et al., 2020). Recently, *CDPK* in ETI and *RPM* and *RAR* in PTI were identified as key genes in the PM resistance of Kentucky bluegrass (Sun et al., 2022). It is unclear whether these genes also play a regulatory role in rust resistance.

Long non-coding RNAs (LncRNAs) can regulate the transcription of downstream genes by activating transcription factors, recruiting them to bind to the promoters of target genes, and promoting their polymerization and expression (Rinn and Chang, 2012; Clark and Blackshaw, 2014; Nejat and Mantri, 2018). LncRNAs also regulate the expression of target genes in Arabidopsis (*Arabidopsis thaliana* L.), melon (*Cucumis melo* L.) and other plants (Severing et al., 2018; Zhou et al., 2020). For instance, a number of lncRNAs with target genes were identified that were resistant to *Fusarium oxysporum* in Arabidopsis (Zhu et al., 2014). Likewise, co-expression analysis of the lncRNAs and target genes were performed in the melon response to PM (Zhou et al., 2020). LncRNAs can regulate the expression of *pathogenesis-related* (*PR*) genes for disease resistance (Seo et al., 2017; Seo et al., 2019). *Arabidopsis* lncRNA *ELENA* can increase the expression level of *PR1* by dissociating the FIBRILLARIN 2/Mediator subunit 19a complex, thereby facilitating resistance against *Pseudomonas syringe* pv. *tomato* DC3000 (Seo et al., 2019). Similarly, lncRNA39026 was shown to induce the expression of the *PR* gene, which increased resistance against *Phytophthora infestans* in tomato (*Solanum lycopersicum* L.) (Hou et al., 2020). *TalncRNA18*, *TalncRNA73*, *TalncRNA106*, and *TalncRNA108* play roles in modulating or silencing the protein-coding gene in response to stripe rust pathogen stress in wheat (Zhang et al., 2013). Furthermore, a total of 22 lncRNAs responding to wheat leaf rust disease were detected using RNA-seq data (Jain et al., 2020). In addition, lncRNAs regulate plant immunity by altering the biosynthesis or signal transduction of plant hormones (Zhang et al., 2018a; Yu et al., 2020). Overexpression of lncRNA ALEX1 was demonstrated to largely upregulate the JA-responsive genes’ expression level to enhance resistance against bacterial blight in rice (*Oryza sativa* L.) (Yu et al., 2020). To date, the regulatory roles of lncRNAs in rust resistance are largely unknown in Kentucky bluegrass.

Kentucky bluegrass has variable polyploidy, aneuploidy chromosome, and high levels or repeat elements in the genome (Bushman et al., 2018). Notably, Kentucky bluegrass cultivars differ in ploidy (Sun et al., 2021). Due to the rapid progress in high-throughput sequencing, the full-length transcriptome has been applied to reveal a unique transcriptome composition and explore the molecular mechanisms under salt stress in perennial grasses (Zhang et al., 2018b; Li et al., 2022). The second-generation sequencing (SGS) technique was used to identify the molecular mechanisms of Kentucky bluegrass in response to low nitrate supply (Sun et al., 2021), drought (Chen et al., 2019), and salt stress tolerance (Bushman et al., 2016). Nevertheless, there is no report using the full-length transcriptome in studying disease resistance in Kentucky bluegrass.

The objective of our study was to evaluate differentially expressed lncRNAs (DELs) with target genes in response to rust using the full-length transcriptome of Kentucky bluegrass. Here, we reported the PacBio transcriptome sequencing of this species. Using this full-length transcriptome as a reference genome, we analyzed the RNA-seq data of rust uninfected and infected plants to explore DELs and DEGs regulation at the transcriptome level. The full-length transcriptome in Kentucky bluegrass was established, which better solved the transcript redundancy problem. The knowledge generated from this study will provide new insights into the molecular mechanisms of rust resistance and aid breeding programs for the development of cultivars of Kentucky bluegrass and other perennial grass species with improved disease resistance.

## 2 Materials and methods

### 2.1 Plant material and pathogen infection

**Figure 1:**
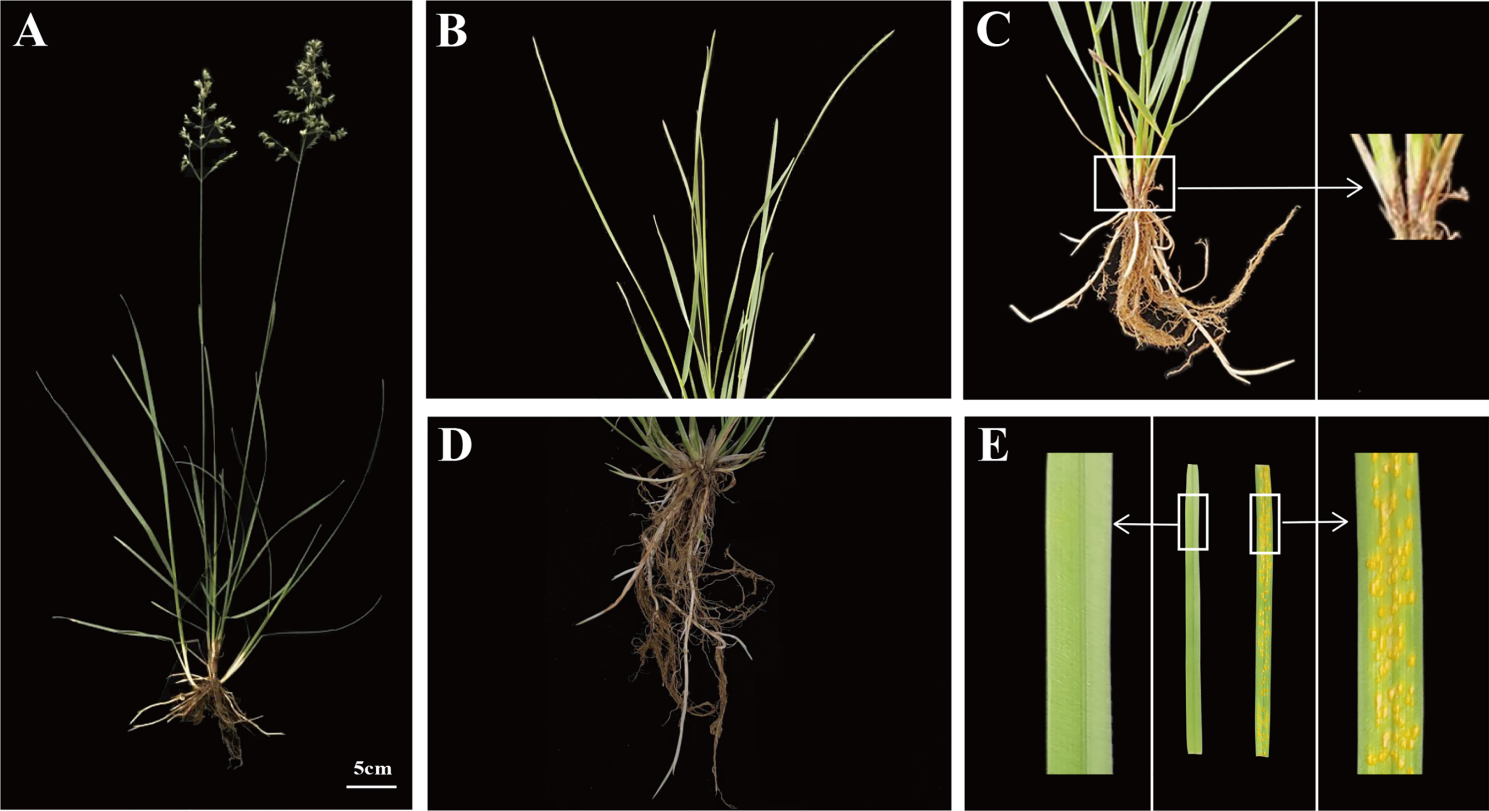
Phenotypes and *P. striiformis*-infected leaves of Kentucky bluegrass cultivar ‘Maoershan’. **(A)** Overall appearance of plants. **(B)** Leaves. **(C)** Stems. **(D)** Roots. **(E)** Mock-inoculated (CK) leaves and rust-infected (RI) leaves. The white boxes represent locally enlarged stem and uninfected and infected leaves.

The Kentucky bluegrass cultivar ‘Maoershan’, originated in the Xing’an Mountains, Heilongjiang Province, China, was used as the test material. The cultivar has been grown in the farm field of Northeast Agricultural University in Harbin, China (126°43’ E, 45°43’ N), for twenty years. The ‘Maoershan’ cultivar has typical characteristics of Kentucky bluegrass, with fine-leaf texture, high shoot density, and outstanding recuperative capacity, etc. (**Figure 1A**).

The plants were cultivated in polyvinyl chloride (PVC) pots filled with 2:1 mixture of sand–vermiculite. The growth conditions in the greenhouse were average temperatures of 24/18 °C (day/night), relative humidity of 60%, and 12 h of illumination (600 μmol·m^-2^·s^-1^) per day. The plants were irrigated with water every 3 days and supplied with a 60 mL of Hoagland solution once a week. After 30 days, three tissues (leaves, stems, and roots) were collected randomly from different plants (**Figures 1B–D**) and immediately stored at -80°C for full-length transcriptome analysis.

Before inoculation with *P. striiformis*, the plants were grown for one day under normal conditions. The individual isolate was purified by ten consecutive generations of *P. striiformis* single-colony inoculation on healthy plants. The purified conidia from one rust patch were sprinkled on the leaves with different paintbrushes. After 21 days, the mock-inoculated leaves and rust-infected leaves were collected (**Figure 1E**) and immediately stored at -80°C for comparative transcriptome analysis. The experiment was a completely randomized design with three replications.

### 2.2 RNA extraction

By using the TRIzol reagent (Invitrogen, United States), the total RNA was extracted from different samples. The quality and purity of the RNA samples were determined by gel electrophoresis and a NanoDrop ND-8000 spectrophotometer (NanoDrop, United States), respectively. The qualified RNA samples were sent to Novogene (Tianjin, China) for sequencing.

### 2.3 Library preparation and sequencing

Using the Clontech SMARTer cDNA Synthesis Kit (TaKaRa, Japan), 5 μg of mixed total RNA was reverse-transcribed into cDNA. The BluePippin Size Selection System (Sage Science, MA) was used for size fractionation and selection. SMRT sequencing libraries were constructed using the Pacific Biosciences DNA Template Prep Kit 2.0. Finally, SMRT cells were sequenced on the Pacific Bioscience RS II platform.

### 2.4 Data analysis of PacBio sequencing reads

The raw sequence data were processed using the SMRTlink 5.0 software to obtain subreads > 50 bp. The subreads were combined and corrected to generate circular consensus sequences (CCSs). The CCSs were classified into full-length and non-full-length sequences according to whether the sequence contained the 5’ ends, 3’ ends, and poly (A) tails. The full-length non-chimeric (FLNC) sequences were obtained by removing poly(A) and linker structures. The clustering of the FLNC sequences was conducted by using the hierarchical n*log(n) algorithm to obtain the consensus sequences. The high-quality FLNC isoforms were obtained after polishing the clustered consensus sequences using Arrow software. Additional nucleotide errors in consensus reads were corrected by the LoRDEC V0.7 software (Salmela and Rivals, 2014). CD-Hit V4.6.8 software was used to remove redundant sequences and ultimately obtain full-length unigenes (Li and Godzik, 2006).

### 2.5 Functional annotation

To analyze the function of the unigenes, we used the BLAST tool (E-value ≤ 10^−10^), Diamond V0.8.36 tool (E-value ≤ 10^−10^), HMMER 3.1 tool (E-value ≤ 10^−3^), and Metascape tool (*p*-value ≤ 0.01) to search the NR, NT, KOG, Swiss-prot, KEGG, GO, and Pfam databases (McGinnis and Madden, 2004; Zhou et al., 2019). The GO annotations were determined by WEGO software (E-value ≤ 10^−5^) according to the best BLASTX hit from the NR database (Ye et al., 2006). KEGG databases (E-value ≤ 10^−5^) and the KEGG automatic annotation server (KAAS) were used for the KEGG pathway analyses (Moriya et al., 2007).

### 2.6 Long non-coding RNAs, simple sequence repeats, and transcription factors analysis

LncRNA candidates were identified by four computational approaches, including the coding potential calculator (CPC) (E-value ≤ 10^−10^), coding potential assessment tool (CPAT) with default parameters, coding–non-coding index (CNCI) with default parameters, and Pfam-scan (E-value ≤ 10^−3^). SSRs in this transcriptome were analyzed using MISA tool (Thiel et al., 2003). ITAK software was used to perform transcription factors (TFs) prediction (Zheng et al., 2016).

### 2.7 Comparative transcriptome of mock-inoculated leaves and rust-infected leaves

On the Illumina HiSeq 6000 platform, the comparative transcriptome libraries were sequenced, and 150-nucleotide-long paired-end sequence reads were produced. After assessing and filtering the transcriptome raw reads, the clean reads were assembled into unigenes by Trinity v2.4.0 (Testone et al., 2019).

### 2.8 Identification of differentially expressed genes resistant to rust

The expression abundance of the unigenes was calculated using the fragments per kilo base of exon per million fragments mapped (FPKM) values (Mortazavi et al., 2008). In addition, the samples of the FPKM values were statistically compared by edgeR software (false discovery rate, FDR ≤ 0.05). KOBAS software (corrected *P-value* ≤ 0.05) was used to perform the KEGG pathway enrichment analysis of the DEGs.

### 2.9 Identification of Long non-coding RNAs resistant to rust

The full-length transcriptome data were used to predict the lncRNAs. The lncRNAs sequences of *Brachypodium distachyon* were obtained from the CANTATAdb 2.0 database (http://cantata.amu.edu.pl) (Szcześniak et al., 2019). The *B. distachyon* genome was downloaded from Ensemble (https://plants.ensembl.org/index.html) and was used to determine the location of the aligned lncRNAs on the genome. In the BioEdit V7.2 software (Borland, United States), the differentially expressed lncRNAs (DELs) sequences of Kentucky bluegrass were used as the request sequences to search the local database of lncRNAs in *B. distachyon*. The DELs by screening cut-offs were *P-value* < 0.05; |log2(FoldChange)| > 1. Subsequently, the coding genes within 10 kb upstream and downstream of the lncRNAs were screened for target gene prediction co-location analysis by Ugene (Unipro UGENE v. 44.0, Russia) (Zhou et al., 2020). The encoded gene was predicted by SoftBerry, and the resulting sequence was annotated by Nucleotide BLAST in NCBI.

### 2.10 Data analysis

SPSS v10.0 software (SPSS Inc, United States) was used for a one-way analysis of variance. The figures were plotted using GraphPad Prism 8 (GraphPad Company, United States) and R v3.4.0 (www.r-project.org). In some figures, the vertical bars represent standard errors (SE). In addition, *t*-tests were used to evaluate the significance of differences at the levels of *P* ≤ 0.05 and *P* < 0.01.

## 3 Result

### 3.1 Overview of the PacBio sequencing datasets

The library was constructed using RNAs extracted from three different tissues of the Kentucky bluegrass cultivar ‘Maoershan’. The SMRT sequencing yielded a total of 29.46 Gb raw data, of which 710,757 were polymerase reads. By removing the adaptor reads and subreads < 50 bp, a total of 15,006,763 subreads (28.37 Gb nucleotides) were identified (**Figures S1A, B**). After self-alignment correction, 626,176 circular consensus sequences (CCSs) with a mean length of 2,299 bp were obtained, among which 565,955 FLNC sequences were successfully extracted (**Table S1**; **Figures S1C, D**). The average length of the FLNC was 2,189 bp (**Table S1**). To obtain the consensus reads, the FLNC sequence was clustered to remove redundancy of the transcripts. After using Arrow software to polish the high-quality consensus reads, a total of 57,558 consensus reads were obtained for subsequent analysis (**Figure S1E**). Finally, 33,541 unigenes with a mean length of 2,233 bp were obtained, and 17,588 of the unigenes were longer than 2,000 bp (**Table S2**; **Figure S1F**).

**Figure 2:**
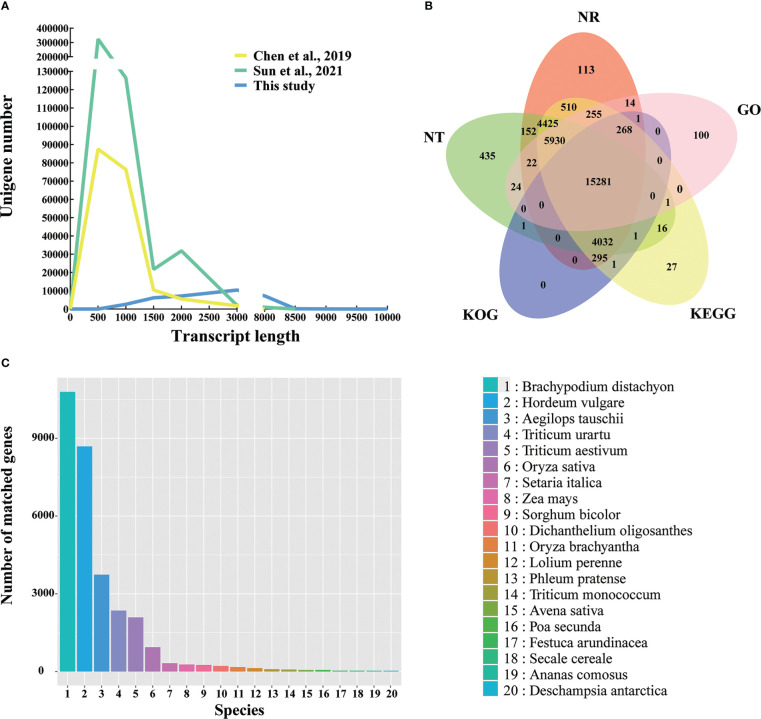
Overview of the Kentucky bluegrass full-length transcriptome dataset. **(A)** Comparison of unigene length in this study and two other studies from second-generation sequencing. **(B)** Venn diagram of NR, GO, KEGG, KOG, and NT database annotation results. **(C)** Species distribution annotated from the NR database.

The Q30 level of the full-length transcripts of Kentucky bluegrass was greater than 93%, which proved that the full-length transcripts have high quality (**Table S3**). Compared with the two SGS projects of Kentucky bluegrass (Chen et al., 2019; Sun et al., 2021), the number of long-length unigenes increased in this study (**Figure 2A**). Previously, the transcript length of the unigenes number peaked at about 500 bp (Chen et al., 2019; Sun et al., 2021); however, the transcript length of this study was about 3,000 bp (**Figure 2A**). The average unigenes length was 718 bp, 747 bp for N50 length, and 374 bp for N90 length in the previous SGS project (Chen et al., 2019), whereas the average unigenes length was 2,233 bp, 2,561 bp for N50 length, and 1,373 bp for N90 length in this study, respectively (**Table S4**). In summary, third-generation sequencing can effectively provide full-length sequences of RNA without short reads and assembly and offer more complete transcriptome data.

### 3.2 Gene annotation

The NR, NT, GO, KEGG, and KOG databases were used to perform the functional annotations of 33,541 unigenes. In all five databases, a total of 15,281 unigenes (45.56%) were shown with high-confidence homologs, and 31,904 unigenes (95.12%) were annotated at least one database (**Figure 2B**). To analyze the conservation of the sequences, the unigenes annotated in the NR database of Kentucky bluegrass were compared with other species. Most of transcripts had a significant level of sequence identity to *B. distachyon*, *Hordeum vulgare*, *Aegilops tauschii*, *Triticum urartu*, and *Triticum aestivum*, which accounted for 34.48%, 27.76%, 11.95%, 7.53%, and 6.68% of the total transcripts, respectively (**Figure 2C**). Previously, the top match species was also *B. distachyon* in the SGS projects, but only accounting for 24.14% of identity (Chen et al., 2019).

**Figure 3:**
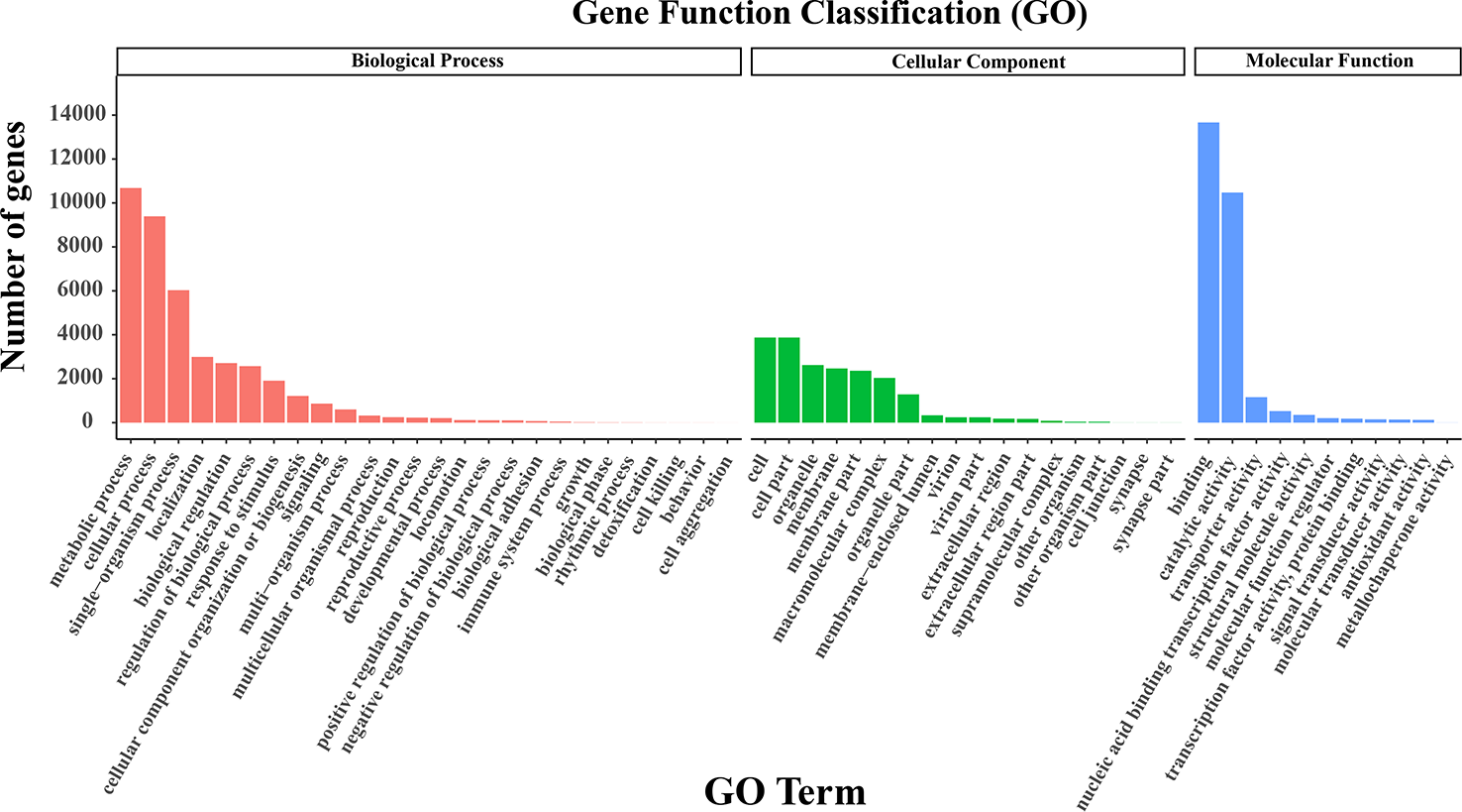
GO classification. The GO terms were classified into biological process, cellular component, and molecular function.

As for GO clustering, the dominant subcategories were ‘cell’ (GO: 0005623) in the cellular component, ‘metabolic process’ (GO: 0008152) in the biological processes, and ‘binding’ (GO: 0005488) in the molecular function category (**Figure 3**). More interestingly, the ratios of unigenes in different GO terms were similar between our research and the two SGS projects of Kentucky bluegrass (Chen et al., 2019; Sun et al., 2021). For KOG categorization, 22,387 unigenes were assigned to 26 groups. The most enriched class was ‘general function prediction only’ (3,975; 17.76%), followed by ‘posttranslational modification’, ‘protein turnover’, ‘chaperones’ (2,598; 11.60%), and then ‘signal transduction mechanisms’ (2,466; 11.02%) (**Figure S2A**). In the KEGG analysis, 19,451 annotated genes were assigned to 44 subcategories in 6 categories. Among them, a few related to signal transduction and carbon metabolism were the most enriched pathways, including ‘signal transduction’ (1,406; 7.23%), ‘carbohydrate metabolism’ (1,379; 7.09%), and ‘translation’ (1,145; 5.87%) (**Figure S2B**).

### 3.3 LncRNAs prediction, simple sequence repeats detection, and transcription factor

**Figure 4:**
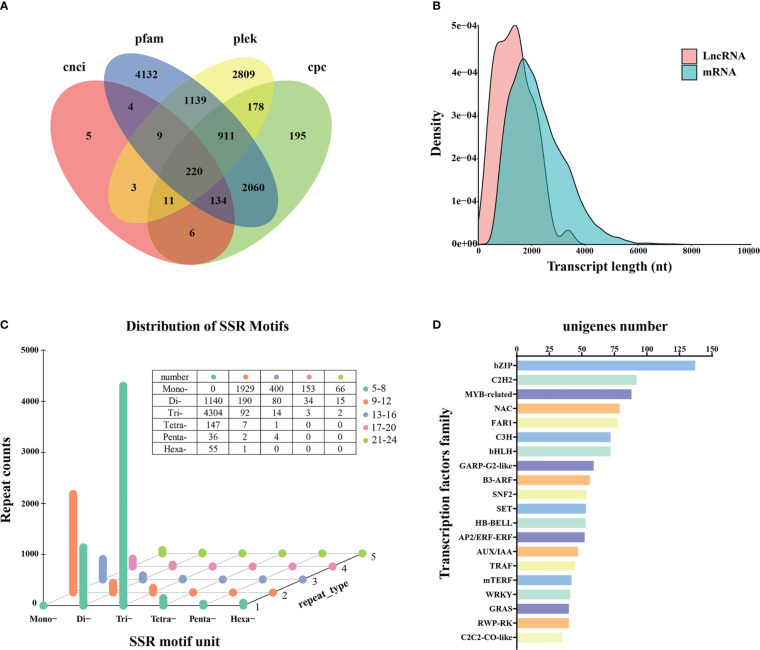
LncRNAs prediction, SSRs detection, and transcription factor analysis. **(A)** LncRNAs prediction. **(B)** Expression density of lncRNAs and protein-coding RNAs (mRNAs). **(C)** SSRs detection. **(D)** Classification of unigenes encoding transcription factors.

A total of 220 transcripts were identified as lncRNAs by four computational methods following annotation (**Table S5**; **Figure 4A**). The average length was 2,239 for mRNAs and 1,359 bp for lncRNAs. Analysis of the expression densities of the lncRNAs and protein-coding RNAs revealed a low expression of long lncRNAs and high expression of short lncRNAs (**Figure 4B**).

A total of 8,675 SSRs were obtained, consisting of one to six tandem repeats (mono-, di-, tri-, tetra-, penta-, and hexa-nucleotide) (**Table S5**). Tri-repeats (4,415; 50.90%) were the most abundant, followed by mono-repeats (2,548; 28.33%) and di-repeats (1,459; 16.82%). The frequencies of tetra-, hexa-, and penta-repeat types only accounted for 1.87%, 0.65%, and 0.48%, respectively (**Figure 4C**).

In addition, 1,604 unigenes were functionally annotated as transcription factors (**Table S5**). The top three common families were 137 bZIP (8.54%), 92 C2H2 (5.74%), and 88 MYB-related (5.45%), respectively (**Figure 4D**). The most frequently represented transcription factor family was C2H2 in the previous SGS projects of Kentucky bluegrass (Chen et al., 2019; Sun et al., 2021), whereas bZIP was the most prominent transcription factor family noted in this study.

### 3.4 Comparative transcriptome analysis of mock-inoculated leaves and rust-infected leaves

#### 3.4.1 Volcano map and KEGG enrichment analysis

**Figure 5:**
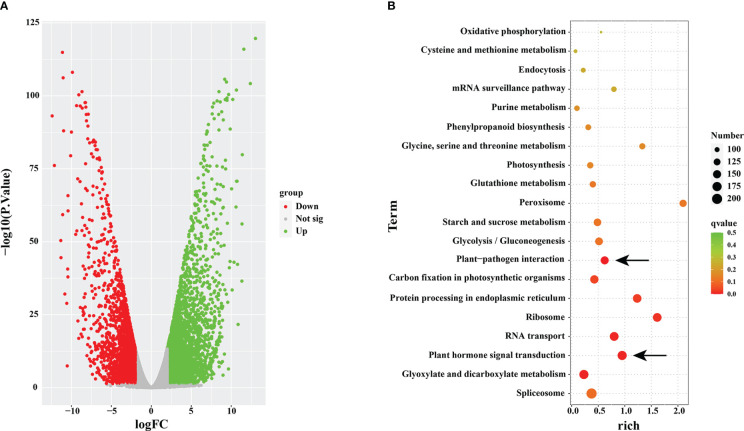
Volcano map and KEGG enrichment analysis. **(A)** Volcano map analysis. **(B)** KEGG enrichment analysis of differentially expressed genes (DEGs) in rust uninfected (CK) and infected (RI) plants.

To examine the accuracy and superiority of full-length transcription, we used this full-length transcriptome as a reference genome to conduct comparative transcriptome analysis in rust uninfected (CK) and infected (RI) plants. A total of 15,711 DEGs were detected in the infected plants, including 8,278 upregulated genes and 7,433 downregulated genes, compared to CK (**Figure 5A**).

To further understand the functions of the DEGs, we conducted the KEGG pathway enrichment analysis. A total of 6,017 DEGs were associated with 119 KEGG pathways, and the most significantly enriched pathway was ‘spliceosome’ (ko03040; FDR= 0.5290) (**Figure 5B**). In addition, ‘plant–pathogen interaction’ (ko04626; FDR= 0.2923) and ‘plant hormone signal transduction’ (ko04075; FDR= 0.6047) were within the top 20 enriched pathways in KEGG pathway analysis (**Figure 5B**).

#### 3.4.2 DELs and DEGs involved in plant hormone signal transduction pathway

After the assembling, annotation, and filtering of all the transcripts from the full-length transcriptome, 220 lncRNAs were screened in Kentucky bluegrass. A total of 105 DELs were found between the rust uninfected and infected plants. A total of 30 DELs were detected after blasting into lncRNAs of *B. distachyon* from the CANTATAdb 2.0 database. After co-location analysis of the coding genes within 10 kb upstream and downstream of DELs was performed, a total of 23 co-expressed DELs were predicted (**Table S6**).

**Figure 6:**
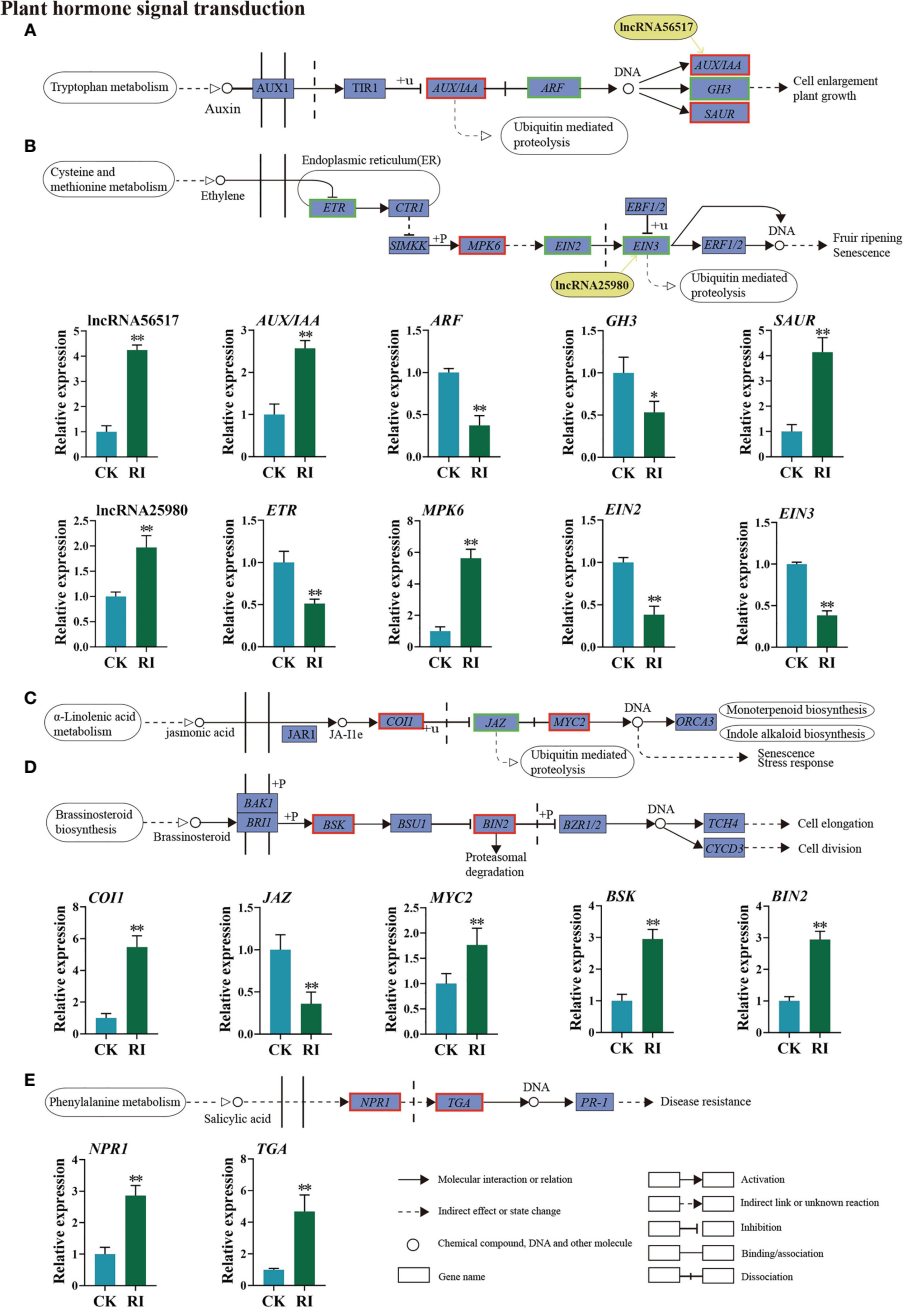
Differentially expressed lncRNAs (DELs) and genes (DEGs) related to phytohormone signaling transduction pathway in the rust uninfected (CK) and infected (RI) plants. **(A)** Auxin (AUX). **(B)** Ethylene (ET). **(C)** Jasmonic acid (JA). **(D)** Brassinosteroid (BR). **(E)** Salicylic acid (SA). The color of the background frame indicates the pattern of gene expression: red for upregulation and green for downregulation. The bars represent standard deviation. Asterisks indicate a significant difference to CK (** *P* < 0.01, * *P* < 0.05).

A list of DEGs and two DELs were identified in the auxin and ethylene signal transduction pathways. Of these genes, lncRNA56517 targeted auxin-related genes, and lncRNA25980 targeted ethylene-related genes. The co-location and expression analysis showed that lncRNA56517 was located in the upstream of the target gene *AUX/IAA* and lncRNA25980 in the upstream of the target gene *EIN3*. Compared with CK, lncRNA56517 and *AUX/IAA* were upregulated 4.25- and 2.57-fold in the infected plants, respectively (**Figure 6A**). The expression pattern was consistent between lncRNA56517 and *AUX/IAA*. LncRNA25980 was upregulated 1.97-fold after infection, whereas the expression level of its target gene *EIN3* showed the opposite trend (0.38-fold) (**Figure 6B**), showing the inconsistent expression pattern between lncRNA25980 and *EIN3*. Additionally, the genes associated with auxin, such as *auxin response factor* (*ARF*) and *auxin-responsive Gretchen Hagen 3* (*GH3*), and those associated with ethylene, such as *ethylene receptor* (*ETR*) and *ethylene insensitive 2* (*EIN2*), were downregulated 0.37-, 0.53-, 0.51-, and 0.38-fold after infection, respectively (**Figures 6A, B**). However, *small auxin-up RNA* (*SAUR*) and *mitogen-activated protein kinases 6* (*MPK6*) genes were significantly upregulated 4.13- and 5.62-fold, respectively (**Figures 6A, B**).

In the plant hormone signal transduction pathway, the DEGs not targeted by lncRNAs were classified into three distinct signal transduction pathways, including the JA, brassinosteroid, and SA signaling pathways. When encountering *P. striiformis* infection, the expression levels of *coronatine insensitive 1* (*COI1*), *myelocytomatosis protein* (*MYC2*), *brassinosteroid signaling kinase* (*BSK*), *brassinosteroid insensitive 2* (*BIN2*), *TGA transcription factors* (*TGA*), and *non-expressor of pathogenesis-related genes 1* (*NPR1*) genes were upregulated (**Figures 6C–E**), while the expression level of the *Jasmonate ZIM-domain* (*JAZ*) gene involved in JA signaling was downregulated (**Figure 6C**).

#### 3.4.3 DELs and DEGs involved in plant–pathogen interaction pathway

**Figure 7:**
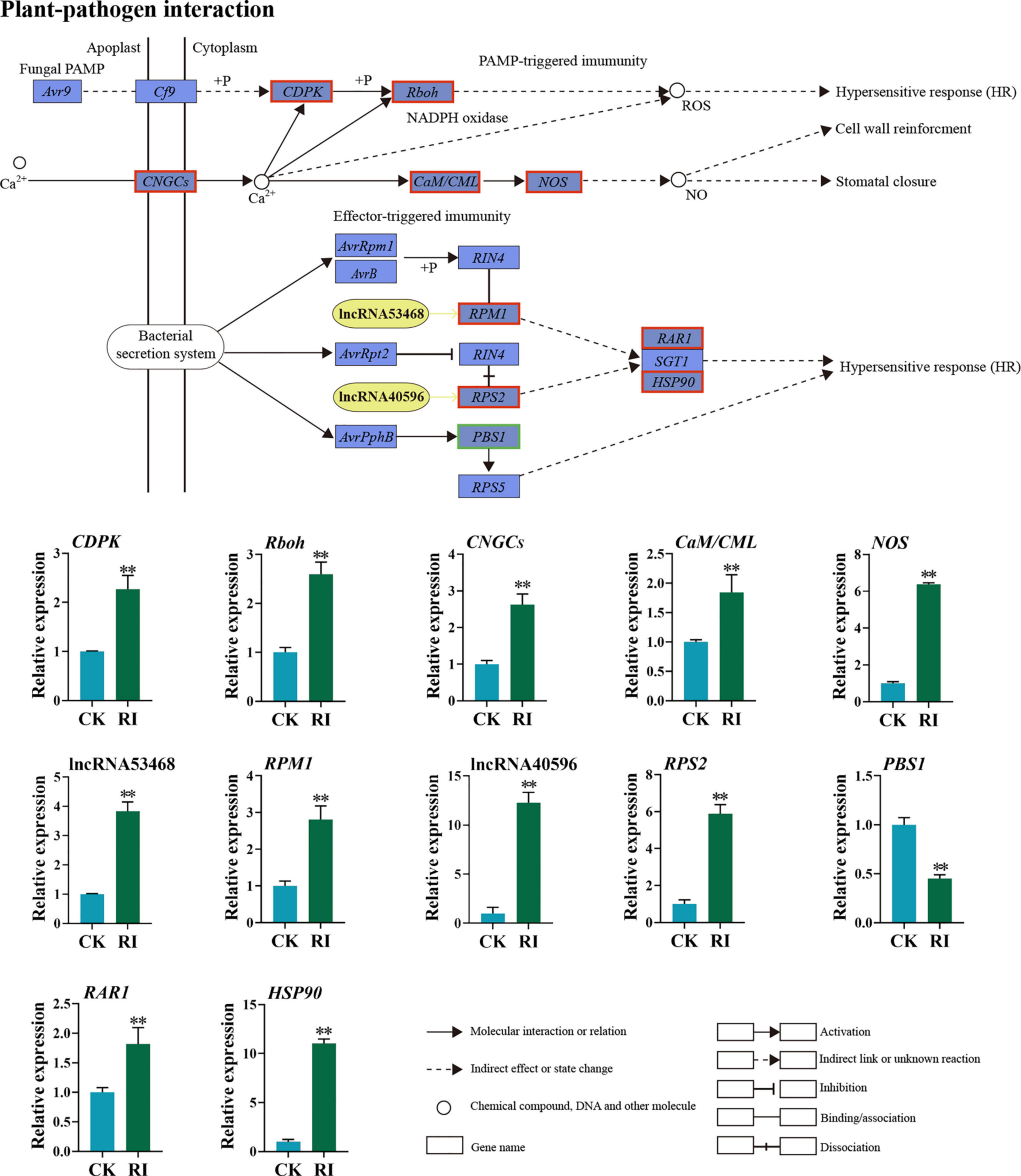
Differentially expressed lncRNAs (DELs) and genes (DEGs) related to the plant–pathogen interaction pathway in the rust uninfected (CK) and infected (RI) plants. The color of the background frame indicates the pattern of gene expression: red for upregulation and green for downregulation. The bars represent standard deviation. Asterisks indicate a significant difference to CK (** *P* < 0.01).

The expression pattern of the DELs and related target genes in the plant–pathogen interaction pathway was detected. Through the co-location and expression analysis, two DELs (lncRNA53468 and lncRNA40596) were predicted. LncRNA53468 was located in the upstream of the target gene *RPM1* (**Figure 7**). Compared with the uninfected control, lncRNA53468 and *RPM1* were upregulated 3.84- and 2.81-fold in the infected plants, respectively (**Figure 7**). LncRNA40596 was located in the upstream of its target gene *RPS2*. The expression level of lncRNA40596 in the infected plants was increased 12.28-fold, and its target gene *RPS2* showed a similar trend (5.90-fold) (**Figure 7**). Compared to the control, *CDPK*-encoding calcium-dependent protein kinases, *CNGCs*-encoding cyclic nucleotide gated channel, *Rboh*-encoding respiratory burst oxidase, *CaM/CML*-encoding calmodulin, and *NOS-*encoding nitric oxide synthase were all highly expressed in the infected plants in the PTI pathway (2.27-, 2.60-, 2.63-, 1.85-, and 6.38-fold, respectively) (**Figure 7**). Furthermore, *RAR1*-encoding retinoic acid receptor and *HSP90*-encoding heat shock protein were significantly elevated in the ETI pathway, in which the expressions were 1.81-fold and 11.04-fold higher in the infected plants than in the control, respectively. In contrast, the *PBS1* gene was downregulated 2.21-fold (**Figure 7**).

## 4 Discussion

Rust is one of the most destructive fungal diseases, which seriously reduces the turf quality and can potentially cause huge economic losses (Bhardwaj et al., 2021). At present, the molecular mechanisms underlying the defense system of Kentucky bluegrass in response to rust remain unclear. Meanwhile, the genetic complexity of this species makes it difficult to mine rust resistance genes. In this study, through sequencing a mixture sample of three different tissues with SMRT from PacBio, the full-length transcriptome of the Kentucky bluegrass dataset was obtained comprising 33,541 unigenes (**Table S1**). Compared with the previous SGS project of Kentucky bluegrass, the average length of unigenes, N50 length, and N90 length were all greatly improved in our study (**Table S4**). Additionally, a total of 220 lncRNAs and 8,675 SSRs were identified (**Table S5**). Our results also demonstrate that using third-generation sequencing to obtain the full-length sequences is more accurate in identifying the lncRNAs’ and unigenes’ length (Roberts et al., 2013; Hackl et al., 2014; Chen et al., 2019; Sun et al., 2021). This allowed us to further explore the lncRNAs’ regulatory mechanisms in response to rust by comparing the full-length transcriptome available in Kentucky bluegrass endogenous hormones, which play crucial roles in regulating plant immune responses (Bari and Jones, 2009; Robert-Seilaniantz et al., 2011). The plant hormone signal transduction pathway was highlighted through the KEGG analysis (**Figure 5B**), with 15 DEGs identified as belonging to the plant hormone signal transduction pathway (**Figure 6**). JA has a positive regulatory effect on necrotizing pathogens in plant immunity (Tsuda and Somssich, 2015). JAZ proteins negatively regulate JA signaling by repressing MYC2 proteins (Zhao et al., 2022). In this study, the upregulation of the *COI1* and *MYC2* genes and downregulation of the *JAZ* gene indicated that JA signal transduction is a key regulator of immunity against *P. striiformis* in Kentucky bluegrass (**Figure 6C**). The BIN2 plays a negative role in the brassinosteroid signaling pathway (Peng et al., 2010). We found that *BIN2* was sharply upregulated in the infected plants (**Figure 6D**), indicating that *P. striiformis* may inhibit brassinosteroid signaling through overexpression of the *BIN2* gene. The role of brassinosteroid in the interaction between Kentucky bluegrass and *P. striiformis* needs to be further clarified. In the SA-mediated signal transduction pathway, *NPR1* and *TGA* were dramatically increased in response to *P. striiformis* infection (**Figure 6E**). This finding was also consistent with the study in wheat that demonstrated that overexpression of *AtNPR1* enhances resistance to *Fusarium graminearum* (Makandar et al., 2006).

Many molecular events are activated to reprogram plants to resist pathogens during plant–pathogen interaction (Allardyce et al., 2013; Nejat et al., 2017). As a general secondary messenger, Ca^2+^ has important functions in the signal transduction pathway of plant disease resistance (Yuan et al., 2017). In this study, numbers of Ca^2+^-related DEGs were markedly upregulated after rust infection in PTI, including *CDPK*, *CNGCs*, and *CaM/CML* (**Figure 7**). Our finding was consistent with the previous finding that the overexpression of the *CDPKs* gene contributed to PM resistance in grapevine (Hu et al., 2021a). However, another study has also shown that *CDPK* gene negatively influenced *Valsa pyri* resistance in pear (*Pyrus pyrifolia*) (Duo et al., 2022). These inconsistent conclusions indicate that the roles of CDPKs in plant disease resistance are complex. During the interaction between plants and pathogens, *R* genes are responsible for recognizing effectors secreted by pathogens, thereby triggering a stronger ETI response (Kim et al., 2012; Białas et al., 2018). Previous studies suggested that *RPM1* and *RPS2* play important roles in plant–pathogen interaction (Banerjee et al., 2001; Mackey et al., 2002). In Kentucky bluegrass, *RPM* was considered a key gene in the response to PM (Sun et al., 2022). In wheat, *TaRPM1* and *TaRPS2* were demonstrated to positively contribute to the HTSP resistance to *Pst* (Wang et al., 2020; Hu et al., 2021b).

Accumulating evidence has demonstrated that lncRNAs can regulate plants against biotic stresses by changing the biosynthesis or signal transduction of plant hormones (Yu et al., 2020; Song et al., 2021). In our study, lncRNA56517 and its target gene *AUX/IAA* were mostly expressed at higher levels involved in the auxin signal pathway in the infected plants (**Figure 6A**), which might suppress the auxin signaling pathway to provide resistance to *P. striiformis*. Similarly, when encountering rust invasion, the *AUX/IAA* gene was upregulated, thereby enhancing the resistance of triticale (×*Triticosecale Wittmack*) to *Pst* (Zhao et al., 2022). In addition, the expression levels of the ethylene-responsive genes *ETR*, *EIN2*, and *EIN3* were decreased after rust inoculation (**Figure 6B**). These results indicate that the ethylene signaling pathway plays a pivotal role in regulating the defense response of Kentucky bluegrass rust infection. Similar results have demonstrated that the ethylene-related genes are highly expressed after *Fusarium oxysporum* f. sp. *cucumerinum* infection in cucumber (*Cucumis sativus* L.) (Dong et al., 2020). LncRNA25980 was identified to be located in the upstream of *EIN3*, and it was noteworthy that the expression pattern of lncRNA25980 was inconsistent with its target gene *EIN3* in the ethylene signaling pathway. In rice, the interaction between lncRNAs and the JA signaling pathway can enhance bacterial blight resistance (Yu et al., 2020), while the functions of lncRNAs and other phytohormones in disease resistance are still largely unknown. Thus, the mechanism of disease resistance mediated by lncRNAs and plant hormones is worth further exploring.

LncRNAs can regulate the expression of *R* genes to increase plant resistance against pathogen invasion (Song et al., 2021). In this study, two lncRNAs (lncRNA53468 and lncRNA40596) and their target genes *RPM1* and *RPS2* were significantly upregulated after infection in the plant–pathogen interaction pathway, compared with uninfected plants (**Figure 7**). Moreover, lncRNA53468 and lncRNA40596 showed the same expression pattern with their target genes (**Figure 7**). These results support that lncRNA53468 and lncRNA40596 are not only involved in the response to rust but also affect the expression levels of their target genes *RPM1* and *RPS2* (**Figure 7**). It has been demonstrated that the function of lncRNA53468 and lncRNA40596 is similar to the role of ELENA1 in *Pseudomonas syringe* pv *tomato* DC3000 resistance in Arabidopsis and lncRNA39026 in *Phytophthora infestans* resistance in tomato (Seo et al., 2019; Hou et al., 2020). Taken together, these results manifest the complicated nature of lncRNAs in the regulation of their target genes and in defense signaling pathways.

In summary, we performed the full-length transcriptome analysis of Kentucky bluegrass and identified a total of 33,541 unigenes, including 220 lncRNAs, 1,604 transcription factors, and 8,675 SSRs. Through comparative transcriptome analysis between the rust uninfected and infected plants, the plant–pathogen interaction and plant hormone signal transduction pathways were significantly enriched. Notably, four DELs and some DEGs involved in the above two pathways were identified. More importantly, the research unraveled the regulatory relationship of lncRNA56517, lncRNA25980, lncRNA53468, lncRNA40596 with their target genes *AUX/IAA*, *EIN3*, *RPM1*, *RPS2* in response to rust in Kentucky bluegrass. The identification of these lncRNAs and their target genes lays a foundation for further elucidating the mechanisms of rust resistance of Kentucky bluegrass and other perennial grass species.

## Data availability statement

The original contributions presented in the study are included in the article/**Supplementary Material**. Further inquiries can be directed to the corresponding authors.

## Author contributions

FX and YajC designed the research; XZ, XS, HW, and YL analyzed the data; XZ and XS wrote the manuscript; YJ revised the manuscript; YanC supervised the experiment. All authors contributed to the article and approved the submitted version.
