# Supplementary material for: Mining of long non-coding RNAs with target genes in response to rust based on full-length transcriptome in Kentucky bluegrass
Source: Front Plant Sci. 2023 May 9;14:1158035. doi: 10.3389/fpls.2023.1158035 (PMC10204806; doi:10.3389/fpls.2023.1158035)
Supplement: Supplementary file 1 [file DataSheet_1.docx]

Supplementary Material

# Supplementary Tables and Figures

## Supplementary Tables

**Supplementary Table 1.** Summary of reads after filtering.

| Classification | Number | Minimum length | Maximum length | Mean length | N50 |
| --- | --- | --- | --- | --- | --- |
| CCS | 626,176 | 99 | 14,996 | 2,299 | 2,627 |
| FLNC read | 565,955 | 50 | 14,057 | 2,181 | 2,523 |
| Consensus read | 57,558 | 57 | 10,248 | 2,188 | 2,518 |
| Unigene | 33,541 | 124 | 10,246 | 2,233 | 2,561 |

Note: CCS: circular consensus sequences; FLNC read: full-length non-chimeric read.

**Supplementary Table 2.** Statistics of length frequency distribution before and after removal of redundant transcript.

| Transcripts length interval | <500 bp | 500-1 kbp | 1 k-2 kbp | 2 k-3 kbp | >3 kbp | Total |
| --- | --- | --- | --- | --- | --- | --- |
| Number of transcripts | 190 | 5,032 | 23,120 | 17,562 | 11,654 | 57,558 |
| Number of unigenes | 48 | 2,627 | 13,278 | 10,388 | 7,200 | 33,541 |

Note: percent of transcripts or unigenes (%): percentage of transcripts or unigenes in corresponding to length range (bp).

**Supplementary Table 3.** The Q30 level of the full-length transcripts and comparative transcriptome of Kentucky bluegrass.

| Sample | Clean reads Q30 (%) |
| --- | --- |
| The full-length transcriptome of Kentucky bluegrass | 93.78 |
| CK1 | 93.45 |
| CK2 | 93.67 |
| CK3 | 93.34 |
| RI1 | 93.18 |
| RI2 | 93.49 |
| RI3 | 93.27 |

**Supplementary Table 4.** Comparison of average unigene length, N50, and N90

| Project | Average unigenes length | N50 | N90 |
| --- | --- | --- | --- |
| Second-generation sequencing | 718 bp | 747 bp | 374 bp |
| Third-generation sequencing | 2,233 bp | 2,561 bp | 1,373 bp |

Note: second-generation sequencing from other study, third-generation sequencing from this study.

**Supplementary Table 5.** Gene structure analysis

| Name | Number |
| --- | --- |
| LncRNAs | 220 |
| Simple sequence repeats | 8,675 |
| Transcription factors | 1,604 |

**Supplementary Table 6.** Identification of lncRNAs resistance to rust

| Name | Number |
| --- | --- |
| DEGs between CK and RI | 105 |
| Blasting into lncRNAs from *Brachypodium distachyon* CANTATAdb 2.0 datebase | 30 |
| Co-expressed DELs with DEGs | 23 |

## Supplementary Figures


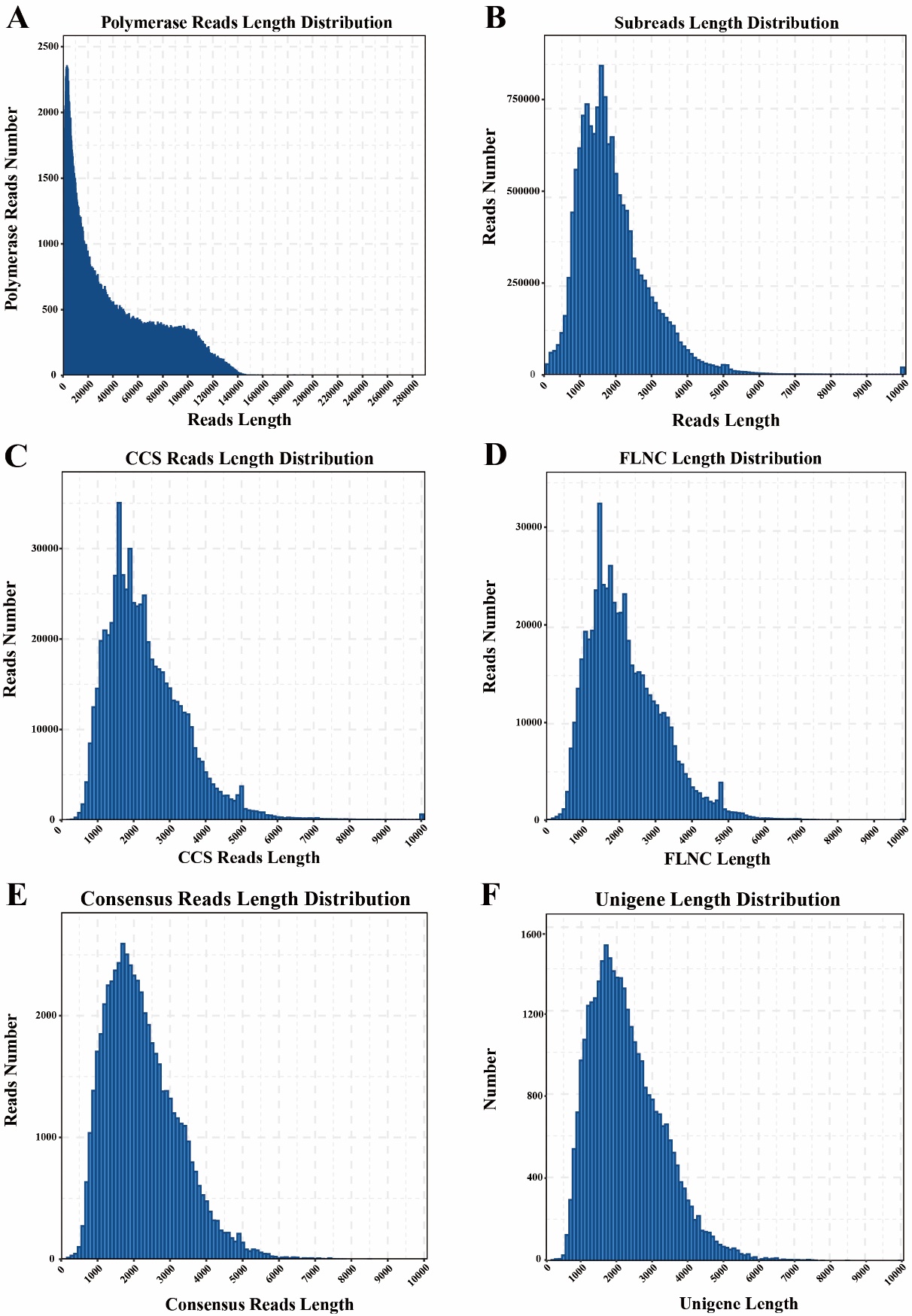


**Supplementary Figure 1.** Reads length distribution during the filtering process. (A) Polymerase reads length distribution. (B) Subreads length distribution. (C) Circular consensus sequence (CCS) reads length distribution. (D) Full-length non-chimeric (FLNC) reads length distribution. (E) Consensus reads length distribution. (F) Unigene length distribution.


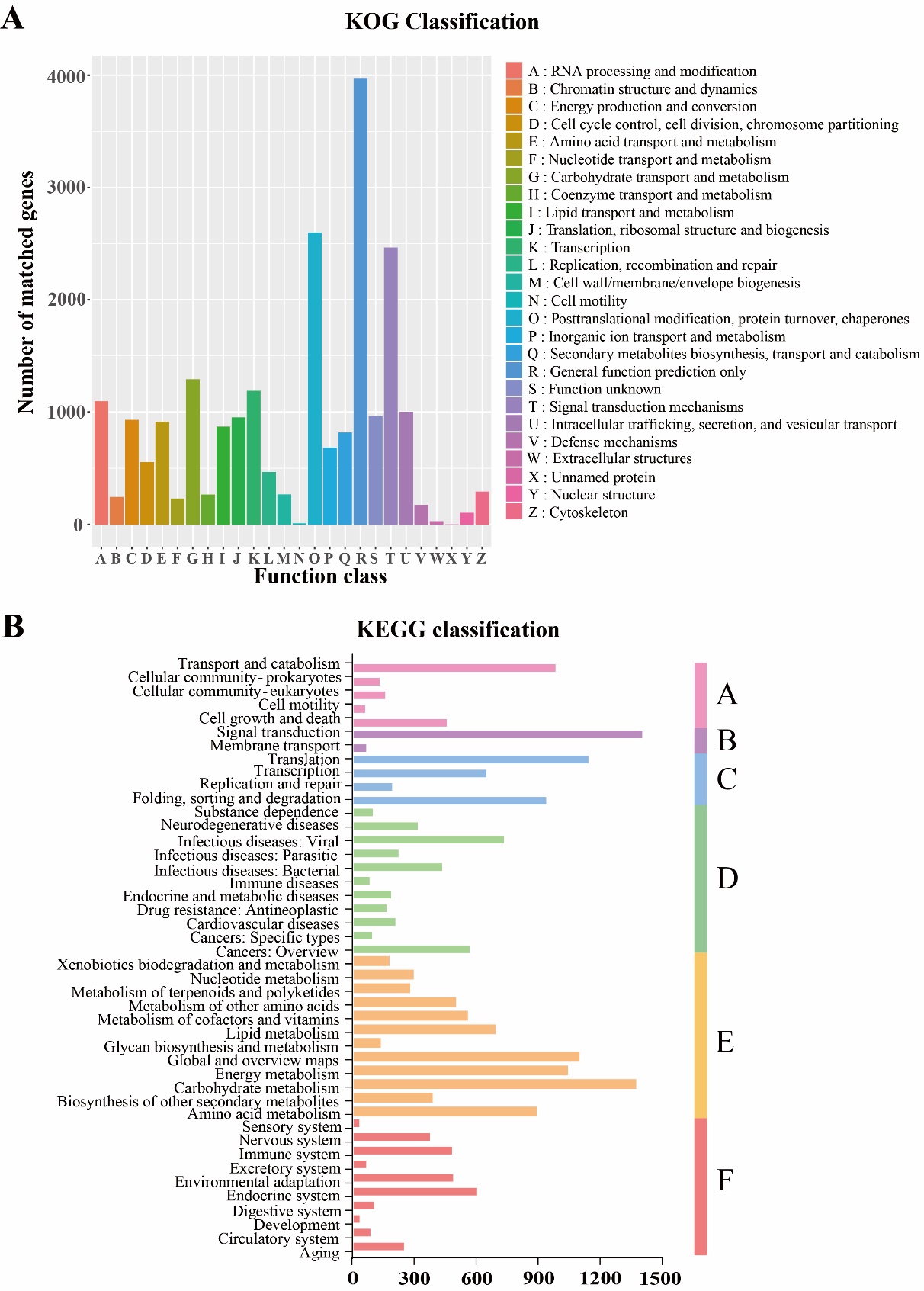


**Supplementary Figure 2.** Unigenes functional classification of the Kentucky bluegrass full-length transcriptome. (A) KOG classification. A-Z respectively represent different biological processes. (B) KEGG classification. A, the cellular process; B, the environmental information processing; C, the genetic information processing; D, the human diseases; E, the metabolism; F, the organismal systems.
